# Acute kidney injury in cancer patients: A nationwide survey in China

**DOI:** 10.1038/s41598-019-39735-9

**Published:** 2019-03-05

**Authors:** Juan Jin, Yafang Wang, Quanquan Shen, Jianguang Gong, Li Zhao, Qiang He

**Affiliations:** 1 Department of Nephrology, Zhejiang Provincial People’s Hospital, Zhejiang, 310014 P. R. China; 2 People’s Hospital of Hangzhou Medical College, Zhejiang, 310014 P. R. China; 3 Department of Respiratory medicine, Sir Run Run Shaw Hospital, Medical School of Zhejiang University, Zhejiang, 310014 P. R. China

## Abstract

Cancer patients have a high risk for acute kidney injury (AKI); however, the incidence, severity, and risk factors of malignancy-related AKI (MR-AKI) are unclear. This study aimed to assess MR-AKI risk factors and provide reliable data for AKI prevention, diagnosis, and management in China. This cross-sectional study analysed data from 44 academic and local hospitals in China. AKI patients were identified based on 2 screening criteria: the 2012 Kidney Disease: Improving Global Outcomes-AKI definition and the expanded screening criteria for patients with no repeated serum creatinine (SCr) test within 7 days and those who recovered from AKI. Patients whose SCr level increased or decreased by 50% during hospitalization, compared with that at admission, were considered to have AKI according to the expanded criteria. A total of 7,604 AKI patients were enrolled (1,418 with MR-AKI). Patient characteristics were compared between the MR-AKI and non-MR-AKI groups. Multivariate logistic models were used to statistically assess risk factors. The proportions of MR-AKI patients in academic and local hospitals were 20.2% and 14.1%, respectively. The incidence of MR-AKI was higher in mid-China (the affluent region), elderly patients, and groups with higher per capita gross domestic product. Among MR-AKI cases, gastrointestinal cancer (50.1%) was the most common malignancy, followed by cancers of the reproductive (15.3%), haematological (13.1%), respiratory (11.8%), and other systems (8.3%), and cancers of unknown classification (1.4%). Of 268 hospital deaths, respiratory, haematological, gastrointestinal, reproductive, other system, and unknown classification cancers accounted for 29.3%, 18.8%, 18.6%, 12.9%, 16.9%, and 20.0%, respectively. Increased age, advanced AKI stage at peak, level of per capita gross domestic product, geographic region, and renal replacement therapy indication were risk factors for hospital mortality in patients with gastrointestinal MR-AKI, whereas cardiovascular disease history, AKI stage at peak, and geographic region were risk factors for mortality in patients with reproductive MR-AKI. The incidence and mortality of MR-AKI vary by hospital, economic level, age, geographic region, and malignancy type. High MR-AKI incidence was associated with gastrointestinal cancers and higher level of medical care provided by academic hospitals in affluent regions such as Beijing, Shanghai, and other provincial-level cities. Elderly patients with advanced gastrointestinal cancer in mid-China showed the highest incidence of MR-AKI and in-hospital mortality, and thus require special attention.

## Introduction

Acute kidney injury (AKI), a common disorder worldwide, is associated with severe morbidity and increased mortality^1^. Cancer patients have a particularly high risk for AKI because they experience multiple renal challenges including malignant cell metastasis and exposures to nephrotoxic drugs and/or radiation. Other conditions, complications, and cancer comorbidities such as increased age, chronic kidney disease (CKD), and cardiovascular disease may also contribute to the susceptibility of patients to kidney injury^2,3^. Although current cancer therapy regimens and rehabilitation care have significantly increased the overall patient survival, these treatments also cause some degree of adverse effects on vital organs such as the liver and kidneys, including causing kidney dysfunction and jeopardizing the quality of life of patients^4^. Because of the high prevalence of AKI in cancer patients and its association with worsened prognosis, identification of the risk factors for AKI and the development of strategies for renal protection during hospitalisation is essential to reduce the associated morbidity and mortality^5^.

The incidence of kidney dysfunction associated with cancer varies greatly among studies and among groups of cancer patients because several factors such as type of malignancy, degree of severity, associated complications, and types of supportive or interventional therapy may contribute to the development of AKI. Christiansen *et al*. reported that patients with kidney cancer, multiple myeloma, and liver cancer had 1-year risks of AKI of 44.0%, 33.0%, and 31.8%, respectively, in a large Danish population^6^, whereas another study detected an AKI rate of 36% in a small number of patients with myelodysplastic syndrome, who had a higher risk than patients with acute myelogenous leukaemia^7^. Critically ill cancer patients were reported to have a 54% risk of AKI^8^. Although these studies provided valuable data for AKI management, few studies have analysed the epidemiology of AKI in a large population of cancer patients from multiple centres nationwide. Although some studies have analysed the risk of AKI with respect to chemotherapeutic agent use^9^; potential nephrotoxins^10^; sepsis, infection, and haematopoietic stem cell transplantation^11,12^; tumour lysis syndrome^13^; age groups^14^; clinical units^15^; and primary malignancy itself^16,17^, these studies were restricted to a single type of malignancy at a single referral centre or had a small number of subjects, such that the overall incidence of AKI in cancer patients and the severity of AKI might be either over- or under-calculated.

We carried out a large-scale national survey and examined 374,286 hospitalized adults in the months of January and July 2013 from 44 hospitals in China, to provide reliable data for a better understanding of the incidence, aetiology, risk factors, treatment, and outcomes of malignancy-related AKI (MR-AKI), and to suggest clinical strategies for preventing malignancy-related renal damage and reducing mortality.

## Results

### Detection rates of MR-AKI were higher in academic hospitals, mid-China, and regions of higher economic level

Because AKI is a common and serious complication of cancer and is associated with increased hospital stay duration and mortality, we analysed the medical records of 7,604 known AKI cases. Among them, 18.6% AKI cases (1,418/7,604) were characterized as MR-AKI. Of the 1,418 MR-AKI patients, 405 (28.6%) were recognized as having AKI according to the 2012 Kidney Disease: Improving Global Outcomes (KDIGO) AKI serum creatinine (SCr) criteria, and the other 1,013 patients (71.4%) were identified as having AKI per the expanded criteria. More than half of the MR-AKI cases (57.5%, 815/1,418) were hospital-acquired AKI, and the remaining cases (42.5%, 603/1,418) were community-acquired AKI.

**Figure 1:**
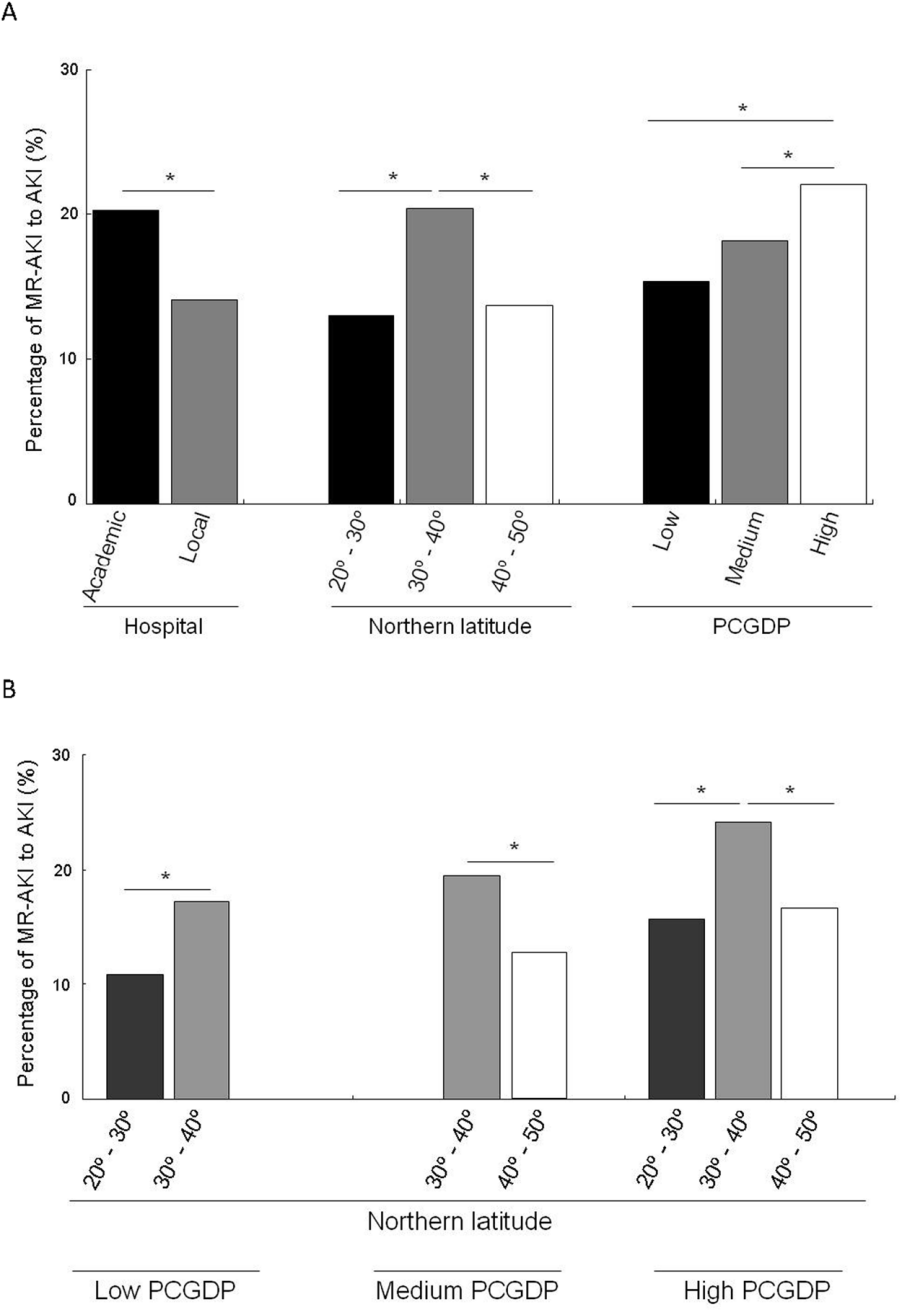
Characteristics of patients with malignancy-related acute kidney injury (MR-AKI) according to hospital level, geographic region, and economic development level. (**A**) Comparison of MR-AKI incidence in terms of hospital level, northern latitudes, and per capita gross domestic product (PCGDP) levels. (**B**) MR-AKI incidence in the northern latitude was stratified by economic development levels (PCGDP).

We noted from our study that the proportion of MR-AKI was significantly higher in academic hospitals than in local hospitals (20.2% vs. 14.1%, *P* < 0.001). The rate was also much higher in the mid-China regions (northern latitude 30°–40°) than in the south (20°–30°) or northern regions (40°–50°). Further, we observed that the economic development level of the region of origin of patients influences the detection rate of MR-AKI. As shown in Fig. 1A, the MR-AKI detection rate was greater in regions with higher per capita gross domestic product (PCGDP) than in those with medium or low PCGDP. After a further stratified analysis at the same level of PCGDP, the mid-region with a latitude of 30°–40° had a greater MR-AKI detection rate than the southern or northern latitude, and with the same latitude range, the MR-AKI detection rate increased in a PCGDP-dependent manner (Fig. 1B).

### Characteristics of MR-AKI among different malignancy categories

The average age of 1,418 MR-AKI patients admitted into participating hospitals in the months of January and July 2013 was 63 years (63.6 ± 14.7 years), and 70.9% of them were men. Approximately half (47.9%) of the patients had stage 1, 25.4% had stage 2, and the rest had stage 3 AKI. About half of the patients (72/143, 50.3%) were treated with renal replacement therapy (RRT). Among the 1,418 cases of MR-AKI, gastrointestinal cancer (50.1%, 710/1,418) was the most common malignancy, followed by reproductive system cancer (15.3%, 217/1,418), haematological cancer (13.1%, 186/1,418), respiratory system cancer (11.8%, 167/1,418), cancer of other systems (8.3%, 118/1,418), and cancer of unknown classification (1.4%, 20/1,418).

We observed that the age of MR-AKI patients with haematological cancer (56.3 ± 16.7 years) was significantly younger than that of patients with other cancers, and that haematological malignancy was the most common primary cancer that caused MR-AKI in young patients (age 18–39 years). The most common cancer that led to MR-AKI in elderly patients (>60 years old) was respiratory malignancy (79.7% of all patients with respiratory cancers had MR-AKI). Moreover, male cancer patients had a greater tendency to develop MR-AKI than female cancer patients. We also noted that patients with reproductive system cancers had a greater tendency (40.6%) to develop post-renal AKI than other AKI classifications. With respect to the causative factors, we identified chemotherapy as the greatest risk factor for MR-AKI in patients with haematological cancer (40.6%), and sepsis conferred the lowest risk of MR-AKI (4.1%) in patients with reproductive system cancer. Patients with respiratory cancer had the lowest risk of developing stage 3 MR-AKI (15.6%), whereas patients with haematological cancer or cancer of other systems had a relatively lower detection rate of delayed recognition (2.2% and 1.7%, respectively) as compared with patients with other cancers (Table 1). The referral rate of patients with haematological cancer (20.4%) was higher than that of patients with other cancers. The hospital cost was the highest for patients with gastrointestinal cancer (7,569 US$). In addition, MR-AKI patients with reproductive system cancer and cancers of unknown classification more likely had pre-existing CKD at the time of diagnosis and treatment (31.3% and 40.0%, respectively) than patients with other cancers, whereas AKI patients with haematological cancer had a much lower incidence of diabetes history (7.5%) than patients with other types of cancers.

**Table 1:** Characteristics of malignancy-related acute kidney injury in different malignancy categories.

	Total (n = 1418)	Hematologic (n = 186)	Respiratory (n = 167)	Gastrointestinal (n = 710)	Reproductive system (n = 217)	*Other systems* (*n* = 118)	*Unknown classification* (*n* = 20)	*P* value
**Age (years)**	63.7 ± 14.7	56.3 ± 16.7	68.8 ± 12.0	63.8 ± 13.6	66.2 ± 16.0	61.4 ± 14.3	75.2 ± 7.4	0.000
**Age group**								
18–39 years	77 (5.4%)	31 (16.7%)	5 (3.0%)	20 (2.8%)	14 (6.5%)	7 (5.9%)	0	0.000
40–59 years	442 (31.2%)	65 (34.9%)	29 (17.4%)	247 (34.8%)	52 (24.0%)	48 (40.7%)	1 (5.0%)	
60–79 years	691 (48.7%)	77 (41.4%)	102 (61.1%)	345 (48.6%)	106 (48.8%)	47 (39.8%)	14 (70.0%)	
≥80 years	208 (14.7%)	13 (7.0%)	31 (18.6%)	98 (13.8%)	45 (20.7%)	16 (13.6%)	5 (25.0%)	
**Men (n, %)**	1001 (70.6%)	119 (64.0%)	124 (74.3%)	553 (77.9%)	130 (59.9%)	65 (55.1%)	10 (50%)	0.000
**AKI classification**								
Pre-renal	711 (50.1%)	58 (31.2%)	95 (56.9%)	420 (59.2%)	71 (32.7%)	56 (47.5%)	11 (55.0%)	0.000
Intrinsic-renal	377 (26.6%)	98 (52.7%)	44 (26.3%)	165 (23.2%)	31 (14.3%)	35 (29.7%)	4 (20.0%)	
Post-renal	150 (10.6%)	5 (2.7%)	4 (2.4%)	45 (6.3%)	88 (40.6%)	7 (5.9%)	1 (5.0%)	
Unclassified	180 (12.7%)	25 (13.4%)	24 (14.4%)	80 (11.3%)	27 (12.4%)	20 (16.9%)	4 (20.0%)	
**Causative factors**								
Surgery before AKI	242 (17.1%)	6 (3.2%)	18 (10.8%)	149 (21.0%)	36 (16.6%)	31 (26.3%)	2 (10.0%)	0.000
Chemotherapy	264 (18.6%)	83 (44.6%)	38 (22.8%)	85 (12.0%)	35 (16.1%)	21 (17.8%)	2 (10.0%)	0.000
Sepsis	99 (7.0%)	17 (9.1%)	14 (8.4%)	51 (7.2%)	9 (4.1%)	4 (3.4%)	4 (20.0%)	0.033
**AKI stage**								
1	675 (47.6%)	83 (44.6%)	91 (54.5%)	337 (47.5%)	92 (42.4%)	66 (55.9%)	6 (55.9%)	0.001
2	361 (25.5%)	44 (23.7%)	50 (29.9%)	186 (26.2%)	46 (21.2%)	29 (24.6%)	6 (30.0%)	
3	382 (26.9%)	59 (31.7%)	26 (15.6%)	187 (26.3%)	79 (36.4%)	23 (19.5%)	8 (40.0%)	
**Recognition**								
Non-recognition	1156 (81.5%)	150 (80.6%)	140 (84.3%)	593 (83.9%)	160 (74.4%)	103 (88.8%)	10 (50.0%)	0.001
Delayed recognition	56 (3.9%)	4 (2.2%)	8 (4.8%)	29 (4.1%)	12 (5.6%)	2 (1.7%)	1 (5.0%)	
Timely recognition	198 (14.0%)	32 (17.2%)	18 (10.8%)	85 (12.0%)	43 (20.0%)	11 (9.5%)	9 (45.0%)	
**RRT indication**	143 (10.1%)	23 (12.4%)	11 (6.6%)	68 (9.6%)	30 (13.8%)	6 (5.1%)	5 (25.0%)	0.01
**RRT rate (RRT treatment/RRT indication)**	72 (50.3%)	15 (65.2%)	3 (27.3%)	32 (47.1%)	13 (43.3%)	5 (83.3%)	4 (80.0%)	0.093
**Renal referral**	197 (13.9%)	38 (20.4%)	14 (8.4%)	95 (13.4%)	29 (13.4%)	11 (9.3%)	10 (50%)	0.000
**Mortality**	268 (18.9%)	35 (18.8%)	49 (29.3%)	132 (18.6%)	28 (12.9%)	20 (16.9%)	4 (20.0%)	0.004
**AKI recovery**								
Complete recovery	312 (28.7%)	43 (30.1%)	27 (23.7%)	169 (30.9%)	43 (24.3%)	25 (27.2%)	5 (38.5%)	0.245
Partial recovery	338 (31.1%)	41 (28.7%)	38 (33.3%)	158 (28.9%)	72 (40.7%)	26 (28.3%)	3 (23.1%)	
Failed recovery	436 (40.1%)	59 (41.3%)	49 (43.0%)	220 (40.2%)	62 (35.0%)	41 (44.6%)	5 (38.5%)	
**Treatment withdrawal**	259 (18.3%)	34 (18.5%)	33 (20.0%)	138 (19.9%)	31 (14.4%)	18 (15.3%)	5 (26.3%)	0.395
**Hospital stay (days)**	22 (13,37	23 (13,36)	20 (11,37)	23 (14,37)	21 (11,41)	24 (15,37)	15 (7.5, 26)	0.156
**Hospital cost (US$)**	6678 (3158, 12753)	5912 (3095, 13967)	5269 (2494, 11371)	7569 (3659, 13764)	4989 (2461, 10606)	6496 (3484,12016)	6181 (3155,12819)	0.005
**Comorbidity**								
Preexisting CKD	246 (17.3%)	35 (18.8%)	19 (11.4%)	101 (14.2%)	68 (31.3%)	15 (12.7%)	8 (40.0%)	0.000
Hypertension	480 (33.9%)	47 (25.3%)	64 (38.3%)	243 (34.2%)	71 (32.7%)	42 (35.6%)	13 (65.0%)	0.005
CVD	274 (19.3%)	29 (15.6%)	43 (25.7%)	114 (16.1%)	48 (22.1%)	27 (23.1%)	13 (65.0%)	0.000
Diabetes	215 (15.2%)	14 (7.5%)	26 (15.6%)	120 (16.9%)	29 (13.4%)	19 (16.1%)	7 (35.0%)	0.005
**AKI recurrence rate**	87 (6.4%)	19 (10.4%)	7 (4.3%)	37 (5.5%)	15 (7.1%)	6 (5.2%)	3 (15.8%)	0.062

Note: Loss of mortality and treatment information: 22 cases. Loss of recognition of MR-AKI information: 8 cases. AKI recovery was calculated after excluding 268 cases who died during the hospital stay and 64 patients whose outcomes were unknown.

### In-hospital mortality and risk factors of death in MR-AKI patients

The all-cause in-hospital mortality of MR-AKI patients was 18.9% (268/1,418). This incidence might be under-calculated because deaths after discharge were not included. Of the 268 documented in-hospital deaths, 132 patients had gastrointestinal cancers, accounting for 18.6% (132/710); 49 patients had respiratory cancers, accounting for 29.3% (49/167); 35 patients had haematological cancers, accounting for 18.8% (35/186); 28 patients had reproductive system cancers, accounting for 12.9% (28/217); 20 patients had cancer of other systems, accounting for 16.9% (20/118); and 4 patients had cancers of unknown classification, accounting for 20.0% (4/20). The mortality rate of MR-AKI in patients with respiratory cancers was the highest among the 6 categories of cancers (Table 1).

By using multivariate analysis, we found that there were no significant causative factors associated with in-hospital mortality in patients with haematological MR-AKI (Supplementary Table S1). However, the referral rate was a protective factor against mortality in patients with respiratory MR-AKI (Supplementary Table S2). We also noted that increased age, high AKI stage at peak, medium and developed levels of PCGDP, a northern geographic region of origin, and an RRT indication were independent risk factors for in-hospital mortality in patients with gastrointestinal MR-AKI (Table 2), whereas history of cardiovascular disease, advanced AKI stage at peak, and a northern region of origin were risk factors that were significantly associated with mortality in patients with reproductive system MR-AKI (Table 3).

**Table 2:** Multivariate logistic regression analysis of factors associated with all-cause in-hospital mortality in patients with gastrointestinal malignancy-related acute kidney injury.

Gastrointestinal MR-AKI		
Variables	OR (95% CI)	*P* value
Age (per 10 years)	1.441 (1.203, 1.727)	0.000
Sex (male vs. female)	1.008 (0.613, 1.658)	0.975
History of CKD (yes vs. no)	0.708 (0.377, 1.328)	0.282
History of CVD (yes vs. no)	1.699 (0.971, 2.973)	0.063
History of hypertension (yes vs. no)	1.272 (0.786, 2.058)	0.327
History of diabetes (yes vs. no)	1.257 (0.737, 2.147)	0.401
**AKI stage at peak**		
1	Reference	
2	2.434 (1.422, 4.169)	0.0001
3	3.285 (1.845, 5.851)	0.000
**PCGDP**		
Low	Reference	
Medium	0.296 (0.156, 0.563)	0.000
High	0.581 (0.344, 0.980)	0.042
**Latitude**		
South China	Reference	
Middle China	0.863 (0.447, 1.666)	0.660
North China	3.171 (1.234, 8.144)	0.017
**Delayed recognition vs. timely recognition**		
non-recognition vs timely-recognition	2.529 (0.950, 6.729)	0.063
RRT indication (yes vs. no)	2.077 (1.036, 4.164)	0.039
Renal referral (yes vs. no)	0.823 (0.420, 1.614)	0.571
**Whether surgery or chemotherapy**		
Neither	Reference	0.192
Surgery	1.359 (0.704, 2.621)	0.361
Chemotherapy	—	0.999
Surgery + chemotherapy	—	—

AKI: acute kidney injury. CKD: chronic kidney disease. CVD: cardiovascular disease. PCGDP: per capita gross domestic product. RRT: renal replacement therapy.

**Table 3:** Multivariate logistic regression analysis of factors associated with all-cause in-hospital mortality in patients with reproductive system malignancy-related acute kidney injury.

Reproductive system MR-AKI		
Variables	OR (95% CI)	*P* value
Age (per 10 years)	1.558 (0.993, 2.446)	0.054
Sex (male vs. female)	1.280 (0.380, 4.312)	0.690
History of CKD (yes vs. no)	0.688 (0.225, 2.102)	0.512
History of CVD (yes vs. no)	4.152 (1.094, 15.759)	0.036
History of hypertension (yes vs. no)	0.598 (0.174, 2.061)	0.416
History of diabetes (yes vs. no)	0.679 (1.203, 1.727)	0.576
**AKI stage at peak**		
1	Reference	
2	3.550 (0.959, 13.134)	0.058
3	4.866 (1.144, 20.7)	0.032
**PCGDP**		
Low	Reference	
Medium	0.384 (0.101, 1.462)	0.161
High	0.332 (0.086, 1.274)	0.108
**Latitude**		
South China	Reference	
Middle China	4.756 (0.600, 37.714)	0.140
North China	25, 129 (1.669, 378.275)	0.020
**Delayed recognition vs. timely recognition**		
non-recognition vs timely-recognition	1.233 (0.199, 7.630)	0.822
RRT indication (yes vs. no)	2.579 (0.704, 9.441)	0.153
Renal referral (yes vs. no)	1.011 (0.260, 3.937)	0.987
**Whether surgery or chemotherapy**		
Neither	Reference	
Surgery	3.328 (0.922, 12.010)	0.066
Chemotherapy	1.540 (0.265, 8.945)	0.630
Surgery + chemotherapy	—	1.000

AKI: acute kidney injury. CKD: chronic kidney disease. CVD: cardiovascular disease. PCGDP: per capita gross domestic product. RRT: renal replacement therapy.

### Renal recovery and factors affecting MR-AKI

Of 1,086 surviving MR-AKI patients, 436 (40.1%) failed to recover from AKI during the hospital stay, whereas the rest of the patients recovered either completely (28.7%) or partially (31.1%) (Table 1). A total of 268 patients died during hospitalization. We excluded 64 cases from the total of 1,418 MR-AKI patients because of missing follow-up information. By using ordinal logistic regression analysis, we observed that RRT indications, low levels of PCGDP, and critical illness were risk factors for renal recovery (Table 4).

**Table 4:** Ordinal logistic regression analysis of factors associated with renal recovery in patients with malignancy-related acute kidney injury.

Variables	OR (95% CI)	*P* value
Age (per 10 years)	0.98 (0.90,1.07)	0.656
Sex (male vs. female)	1.18 (0.91,1.53)	0.208
History of CKD (yes vs. no)	0.97 (0.71,1.34)	0.869
History of CVD (yes vs. no)	0.92 (0.66,1.29)	0.641
History of hypertension (yes vs. no)	0.99 (0.76,1.30)	0.962
History of diabetes (yes vs. no)	1.21 (0.86,1.70)	0.268
**AKI stage at peak**		
1	1.01 (0.73,1.39)	0.972
2	0.97 (0.68,1.38)	0.868
3	Reference	
**PCGDP**		
Low	0.68 (0.50,0.93)	0.016
Medium	0.75 (0.57,0.98)	0.037
High	Reference	
**Latitude**		
South China	1.08 (0.59,1.97)	0.808
Middle China	0.97 (0.68,1.38)	0.882
North China	Reference	
non-recognition vs timely-recognition	0.79 (0.52,1.19)	0.256
Delayed recognition vs. timely recognition	1.42 (0.68,2.97)	0.357
RRT indication (yes vs. no)	1.67 (1.01,2.75)	0.046
Renal referral (yes vs. no)	1.09 (0.72,1.64)	0.685
Critical illness	1.35 (1.16,1.70)	0.013
**malignant tumor site**		
Hematologic	0.99 (0.69,1.42)	0.935
Respiratory: branches, lung	1.26 (0.86,1.84)	0.243
Gastrointestinal	Reference	
Reproductive system and Urinary tract	1.29 (0.90,1.85)	0.170
Central nervous system	0.94 (0.43,2.04)	0.878
Others	1.62 (0.99,2.67)	0.056

AKI: acute kidney injury. CKD: chronic kidney disease. CVD: cardiovascular disease. PCGDP: per capita gross domestic product, Low: <33,000 Yuan, medium: 33,000–58,000 Yuan, and high or influent region such as Beijing and Shanghai and other provincial cities: >58,000 Yuan. RRT: renal replacement therapy.

## Discussion

AKI is a global health problem^18–20^ and a well-known complication of cancer. The incidence of AKI in hospitalized cancer patients seems to be increasing because of aggressive cancer therapies^21^. The detection rate of AKI is sometimes underestimated if the recognition of AKI is based on administrative data such as International Classification of Diseases 10th revision codes, in which case the disease may be unrecognized or undocumented^22^. To overcome the limitation of the current AKI definitions, in this study, we included both the KDIGO criteria and the expanded criteria for AKI screening. By using the expanded criteria, we identified 1,013 AKI cases (71.4%) in addition to 405 patients (28.6%) recognized as having AKI according to the KDIGO standard screening protocol. We found that 18.6% of AKI patients were characterized as having MR-AKI. With the combination of the 2 screening approaches, our detection rate was significantly increased and the data were comparable to previous reports^6^. The incidence of AKI in various cancers has been reported to be in a range of 12–49%^16,23–26^. We observed the highest risk of AKI in gastrointestinal cancer patients, whereas the incidence of haematological MR-AKI was lower than that reported by Khalil *et al*., who found a 31.8% incidence of AKI in lymphoma patients^27^. This considerable discrepancy could be explained by the survey scale, subtype and stage of cancer, severity of illness, and regions of origin of the patients. To our best knowledge, this is the largest survey of AKI in pooled multiple cancer patients. Our study revealed that the MR-AKI detection rate of 18.6% in hospitalized patients was more reliable and likely represents the actual incidence of MR-AKI in China. Thus, the overall detection rate of MR-AKI in the 374,286 hospitalized adults enrolled in our survey in 2013 was 0.37%. On the basis of this incidence, we estimated that there were approximately 540,000 hospitalized adults with MR-AKI in China in 2013.

In this study, we found a higher detection rate of MR-AKI in the medium-north region than in the south and far north regions. It was reported that air pollution was more severe in the medium-north region than in the south and far north regions, and that long-term exposure to elevated levels of particulate matter of <2.5 μm diameter was associated with an increased risk of membranous nephropathy^28^. Whether environmental factors, including food and water pollution, contribute to the MR-AKI incidence needs to be further investigated in the future.

We also demonstrated that patients with haematological cancer had a relatively low rate of delayed recognition (2.2%), whereas the referral rate of patients with haematological cancer (20.4%) was higher than that of patients with other cancers. Our findings indicate that in patients with haematological cancer, MR-AKI developed and progressed very rapidly, the disease was very serious that AKI could not be diagnosed and managed in a timely manner, and that the physicians in the haematological department did not have sufficient resources to manage the disease.

The cause of AKI in hospitalized cancer patients is multifactorial. Consistent with the findings of previous studies, our study also revealed that malignancy type, advanced age, intensive chemotherapy use, and nephrotoxic drug use were independent risk factors of AKI in cancer patients^5,29,30^. Our study also showed a positive correlation between the detection rate of AKI and the level of PCGDP. The level of PCGDP was significantly associated with increased rates of referral and hospital admission of MR-AKI patients in the region. Compared with patients from poorly developed regions in terms of PCGDP, those who came from regions with high levels of PCGDP received a more timely diagnosis, were more likely to have received appropriate RRT, and stayed on the course of treatments. A recent global meta-analysis of AKI published by Mehta *et al*. found that countries that spent more gross domestic product (GDP) on total health-care expenditure had a higher detection rate of AKI and a lower rate of AKI-associated mortality than those that spent less GDP on health care^29,31,32^. Their findings suggested that the AKI incidence was associated with economic development and advanced treatments such as novel, expensive anti-cancer drugs and therapeutic approaches in developed countries. In agreement with their report, our study showed a similar association of GDP with the AKI incidence, but a mixed result for the all-cause in-hospital AKI mortality. We found that patients from intermediately developed regions had a lower risk for all-cause in-hospital mortality and lower rates of treatment discontinuation, whereas those from well-developed regions had a higher risk for all in-hospital mortality than patients who came from poorly developed regions. As treatment withdrawal was less common in well-developed regions, we assumed that more severely ill patients with good income might have remained hospitalized for treatment, which contributed to their higher in-hospital mortality, whereas severely ill patients in poorly developed regions might have died after discharge. It should be noted that patients with low income or extreme financial hardship might die after discharge. Accordingly, our study found that the high incidence of MR-AKI was found to be associated with the higher level of medical care provided by academic hospitals in affluent regions, and with gastrointestinal cancers. A possible explanation is that wealthy patients who were in critical condition were more likely to be admitted to large, prominent hospitals for treatment, and patients with gastrointestinal cancers were treated more aggressively with chemotherapy. Our data revealed the grim health situation of MR-AKI patients in poor regions in China, where the disease burden was high, the medical referral rate was low, the diagnosis was often delayed, and the mortality rate was underestimated. Thus, these regions need urgent attention from government health-care agencies.

This study has several limitations. First, the study included only subjects with repeated SCr tests. Potential AKI patients with only 1 SCr test, as well as those whose kidney function had already plateaued during hospitalization, were excluded from the study. Second, the screening criteria used in this study were based on changes in SCr levels rather than urinary output. Although the urinary output criteria might have allowed us to identify more AKI cases, results based on the creatinine criteria were less affected by diuretics use than those based on the urine output^33^. Third, the presence and aetiology of AKI in this study were determined according to medical records. Therefore, AKI patients with incomplete documents might have been missed. Further studies are needed to compare the MR-AKI incidence with various screening criteria to determine whether AKI patients with a single SCr test should be included or not. Fourth, subgroups of MR-AKI were not stratified by chemotherapy, hypoalbuminaemia, and nephrotoxins such as angiotensin-converting enzyme inhibitors, angiotensin receptor blockers, non-steroidal anti-inflammatory drugs, antibiotics, or traditional Chinese herbal medicine, as these data were unavailable although such factors are related causes of AKI. In addition, as patients were normally treated with a combination of multiple medications or exposed to multiple nephrotoxins, it was impossible to stratify and determine which agent contributed to AKI development. Lastly, large-scale prospective studies are needed to confirm our findings.

## Methods

### Participants and survey protocol

The survey protocol has been described in detail elsewhere^34^. In the months of January and July 2013, a total of 374,286 adult patients (≥18 years old) from 22 academic hospitals and 22 local hospitals in mainland China were screened for suspected AKI based on changes in their SCr levels documented by the Laboratory Information System. We retrieved the hospital medical records of suspected AKI patients who were hospitalized during 2 independent months, January and July 2013, to confirm the diagnosis of AKI. A total of 7,604 AKI patients were confirmed and enrolled for the survey.

AKI was diagnosed according to the 2012 KDIGO-AKI SCr definition^35^. Briefly, AKI was diagnosed in the presence of one of the following laboratory results: (i) the SCr level increased by >26.5 μmol/L (0.3 mg/dL) within 48 h; (ii) the SCr level increased by ≥1.5 times over the baseline level, and the same result could be obtained again or predicted within 7 days; and (iii) the urine volume had been <0.5 mL/(kg.h) for ≥6 h. For patients with no repeated SCr test within 7 days and those who had recovered from AKI, we expanded the definition criteria to an increase or a decrease in SCr level by 50% during the hospital stay, as compared with the baseline SCr level at admission. Patients with pre-existing stage 5 CKD or those who had undergone nephrectomy or kidney transplantation were excluded. Patients who had a peak SCr level of <0.6 mg/dL or those with fluctuating SCr levels that were not attributed to AKI were also excluded from the study^36^.

For all AKI cases detected by the survey, medical records were retrieved to document the socio-demographic status, comorbidities, hospitalization data, diseases or conditions that could cause renal hypoperfusion (hypovolaemia or cardiorenal syndrome), nephrotoxic medications or other nephrotoxins, critical illnesses, AKI classification, AKI stage^37^, RRT indications^38,39^ and modalities, renal referral, all-cause in-hospital death, and renal recovery at discharge. The study was approved by the Ethics Committee of Zhejiang Provincial People’s Hospital, and informed consent was obtained from all participants. The medical records were obtained in an anonymous manner.

### Groups and definitions

Identified AKI patients with a primary diagnosis of cancer at discharge or death were classified as having MR-AKI, and the others were classified as having non-MR-AKI. MR-AKI patients were further divided into 8 categories based on tumour origin: haematological (leukaemia, malignant lymphoma, and multiple myeloma), respiratory (bronchi and lungs), gastrointestinal (oesophagus, stomach, intestine, colon, and rectum), other digestive (liver, gallbladder, and pancreas), reproductive (female and male), central nervous, urinary tract (kidney, ureter, and bladder), and rare cancers.

Critical illness was defined as any of the following conditions: sepsis, multiple organ dysfunction, shock, disseminated intravascular coagulation, acute respiratory distress syndrome, and endotracheal intubation ventilator therapy. The indications for RRT were defined as volume overload unresponsive to diuretic therapy, severe hyperkalaemia (≥6 mmol/L), metabolic acidosis (pH ≤7.3), blood urea nitrogen levels ≥60 mmol/L, and overt uremic manifestations such as pericarditis and encephalopathy^40^.

We defined AKI as any documentation of increased SCr level, renal dysfunction, decrease in urine output, or treatment adjustments such as discontinuation of nephrotoxic medications based on the KDIGO or KDIGO expanded criteria, as described above. We defined timely recognition as detection of AKI by physicians within 3 days without an indication of progression to a higher stage; otherwise, we considered the AKI case to have delayed recognition. We defined an AKI case as ‘non-recognition’ if a patient who met the AKI screening criteria was not diagnosed as having AKI by the physician in charge during hospitalization. Treatment withdrawal because of extreme financial hardship or serious illness was also documented. The details of the study design and work process were described previously^34^.

The recovery of kidney function was defined as previously reported^41^. We defined full recovery as a decrease in the SCr level to below the threshold SCr level, which was ±10% of the baseline SCr level. We defined partial recovery as a decrease in the SCr level by ≥25% from the peak level but remaining above the threshold level and with a requirement for dialysis or a decrease in the SCr level by <25% from the peak level.

### Statistical analysis

The proportion of MR-AKI cases among all AKI cases was calculated and stratified by hospital type (academic vs. local), geographical region (southern, middle, and northern China), and level of economic development (PCGDP), as reported previously^34^. The clinical characteristics of MR-AKI patients were also stratified by hospital type. The clinical characteristics, diagnosis, treatment, and prognosis were compared between MR-AKI and non-MR-AKI patients, and between haematological MR-AKI and non-haematological MR-AKI patients. Categorical variables are presented as counts and proportions. Continuous variables are described as mean values ± standard deviations or median values (interquartile range) as appropriate. Groups were compared using 1-way analysis of variance or Kruskal-Wallis tests for continuous variables, and the chi-square test for categorical variables. Differences between groups were determined using Student’s *t*- and Mann-Whitney *U*-tests. Logistic regression models were used to analyse the association between all-cause in-hospital mortality and relevant covariates. Odds ratios with 95% confidence intervals and *P* values of the Wald chi-square test were reported. The covariates included in the multivariable logistic regression models were age (grouped per 10 years), sex (male vs. female), highest AKI stage, recognition of AKI (non-recognition, delayed recognition, timely recognition), geographical regions, and PCGDP. The following covariates were also included and grouped by yes vs. no: CKD, cardiovascular disease, hypertension, type 2 diabetes mellitus, renal referral, and RRT indications. Epidata software (version 3.1; Epidata Association, Odense, Denmark) was used for data entry and management. All *P*-values were 2-sided, and *P* < 0.05 was considered statistically significant. Analyses were performed using SPSS software (version 19.0; SPSS, IBM, Chicago, IL, USA).

## Supplementary information

Supplementary Table
