# Supplementary material for: Acute kidney injury in cancer patients: A nationwide survey in China
Source: Sci Rep. 2019 Mar 5;9:3540. doi: 10.1038/s41598-019-39735-9 (PMC6401015; doi:10.1038/s41598-019-39735-9)
Supplement: Supplementary file 1 — Supplementary Table [file 41598_2019_39735_MOESM1_ESM.pdf]

Acute kidney injury in cancer patient: a nationwide survey in China

Running title: AKI in cancer patient in China

Juan Jin[1,2], lang_018@163.com

Yafang Wang[3], yf725@126.com

Quanquan Shen[1,2], spring198457@sina.com

Jianguang Gong[1,2], gojigu311@aliyun.com

Li Zhao[1,2], zdf24zl@163.com

Qiang He[1,2*], qianghe1973@126.com

[1]Department of Nephrology, Zhejiang Provincial People's Hospital, Zhejiang 310014, P.R. China

[2]People's Hospital of Hangzhou Medical College, Zhejiang 310014, P.R. China.

[3]Department of Respiratory medicine, Sir Run Run Shaw Hospital, Medical School of Zhejiang University, Zhejiang 310014, P.R. China

***Corresponding author**

Qiang He

Department of Nephrology, Zhejiang Provincial People's Hospital, No.158 Shangtang Road, Xiacheng District, Hangzhou, Zhejiang 310014, P.R. China

People's Hospital of Hangzhou Medical College, No.158 Shangtang Road, Xiacheng District, Hangzhou.Zhejiang 310014, P.R. China

Tel:+86-571-85893689

Fax: +86-571-85893689

Email: qianghe1973@126.com

Supplementary Table 1. Multivariate logistic regression analysis of factors associated with all-cause in-hospital mortality in patients with Hematologic MR-AKI.

Hematologic MR-AKI		
Variables	OR (95% CI)	*P* value
Age (per 10 years)	1.143 (0.862, 1.516)	0.352
Sex (male vs. female)	1.234 (0.516, 2.955)	0.636
History of CKD (yes vs. no)	1.090 (0.377, 3.147)	0.874
History of CVD (yes vs. no)	1.441 (1.203, 1.727)	0.073
History of hypertension (yes vs. no)	1.135 (0.408, 3.159)	0.808
History of diabetes (yes vs. no)	0.390 (0.068, 2.245)	0.292
AKI stage at peak		
1	Reference	
2	0.996 (0.359, 2.767)	0.994
3	1.016 (0.350, 2.943)	0.977
PCGDP		
Low	Reference	
Medium	0.447 (0.122, 1.636)	0.224
High	1.031 (0.368, 2.890)	0.954
Latitude		
South China	Reference	

Middle China	1.857 (0.342, 10.071)	0.473
North China	3.486 (0.290, 1.850)	0.325
Delayed recognition vs. timely recognition		
non-recognition vs timely-recognition	--	0.999
RRT indication (yes vs. no)	1.919 (0.528, 6.975)	0.322
Renal referral (yes vs. no)	1.728 (0.458, 6.523)	0.419
Whether surgery or chemotherapy		
Neither	Reference	
Surgery	0.733 (0.061, 8.754)	0.806
Chemotherapy	0.997 (0.425, 2.336)	0.994
Surgery + chemotherapy	--	1.000

AKI: acute kidney injury. CKD: chronic kidney disease. CVD: cardiovascular disease. PCGDP: per capita gross domestic product. RRT: renal replacement therapy.

Supplementary Table 2. Multivariate logistic regression analysis of factors associated with all-cause in-hospital mortality in patients with Respiratory MR-AKI.

Respiratory MR-AKI		
Variables	OR (95% CI)	*P* value
Age (per 10 years)	1.272 (0.847, 1.910)	0.247

Sex (male vs. female)	1.650 (0.636, 4.281)	0.303
History of CKD (yes vs. no)	1.364 (0.375, 4.958)	0.638
History of CVD (yes vs. no)	1.210 (0.467, 3.136)	0.695
History of hypertension (yes vs. no)	0.971 (0.399, 2.361)	0.949
History of diabetes (yes vs. no)	1.114 (0.400, 3.099)	0.837
AKI stage at peak		
1	Reference	
2	1.807 (0.736, 4.435)	0.197
3	1.181 (0.320, 4.361)	0.803
PCGDP		
Low	Reference	
Medium	0.859 (0.298, 2.478)	0.778
High	0.751 (0.265, 2.128)	0.590
Latitude		
South China	Reference	
Middle China	3.398 (0.815, 14.174)	0.093
North China	2.609 (0.328, 20.744)	0.365
Delayed recognition vs. timely recognition		
non-recognition vs	0.738 (0.083, 6.572)	0.785

timely-recognition		
RRT indication (yes vs. no)	6.372 (0.972, 41.762)	0.054
Renal referral (yes vs. no)	0.071 (0.008, 0.663)	0.020
Whether surgery or chemotherapy		
Neither	Reference	
Surgery	0.343 (0.068, 1.737)	0.196
Chemotherapy	0.734 (0.256, 2.101)	0.564
Surgery + chemotherapy	--	--

AKI: acute kidney injury. CKD: chronic kidney disease. CVD: cardiovascular disease.

PCGDP: per capita gross domestic product. RRT: renal replacement therapy.
